# Optical Limiting Properties of DNA Biopolymer Doped with Natural Dyes

**DOI:** 10.3390/polym16010096

**Published:** 2023-12-28

**Authors:** Petronela Gheorghe, Adrian Petris, Adina Mirela Anton

**Affiliations:** 1 National Institute for Laser, Plasma and Radiation Physics, 409 Atomistilor Street, 077125 Magurele, Romania; anastasiumirela@yahoo.com; 2 Faculty of Chemical Engineering and Biotechnologies, National University of Science and Technology Politehnica Bucharest, 1-7 Polizu Street, 011061 Bucharest, Romania

**Keywords:** eco-friendly materials, DNA biopolymers, natural dyes, nonlinear optical response, optical limiting

## Abstract

The high-power lasers have important implications for present and future light-based technologies; therefore, the protection measures against their high-intensity radiation are extremely important. Currently, a great deal of interest is directed towards the development of new nonlinear optical materials for passive optical limiters, which are used to protect the human eye and sensitive optical and optoelectronic devices from laser-induced damage. Biopolymers doped with natural dyes are emerging as a new class of optical materials with interesting photosensitive properties. In this paper, the optical limiting capability of deoxyribonucleic acid bio-polymer functionalized with Turmeric natural dye has been demonstrated for the first time, to the best of our knowledge. The experimental investigation of the optical limit has been done by the Intensity-scan method in the NIR spectral domain at the important telecommunication wavelength of 1550 nm, using ultrashort laser pulses (~120 fs). Several optical properties of this natural dye are presented and discussed. The values of the optical transmittance in the linear regime, the saturation intensity of the nonlinear transmittance curves, and the coefficient of the nonlinear absorption have been determined. The influence of the DNA biopolymer and natural dye concentration on the optical limiting properties of the investigated biomaterials is reported and discussed. The photostability and thermal stability of the investigated solutions have also been evaluated by monitoring the temporal decay of the normalized absorption spectra under illumination with UVA light and heating, respectively. Our results evidence the positive influence of the DNA, which embeds Turmeric natural dye, on the optical limiting functionality itself and on the photostability and thermal stability of this novel material. The performed study reveals the potential of the investigated novel biomaterial for applications in nonlinear photonics, in particular in optical limiting.

## 1. Introduction

Lately, scientists around the world have been concerned about the protection of the environment and biodiversity by enhancing the sustainability and quality of eco-friendly materials. Natural dyes are emerging as an important class of biodegradable optical materials used for modern applications in optics and photonics, security, and safety and have been intensely studied in recent years due to their interesting photosensitive properties [1,2,3]. The deoxyribonucleic acid (DNA) functionalized with different synthetic dyes, e.g., Rhodamine B, Disperse Red 1 [4,5,6], represents novel materials with practical applications in photonics. Biopolymers doped with natural dyes are emerging as a new class of optical materials with interesting photosensitive properties.

**Figure 1:**
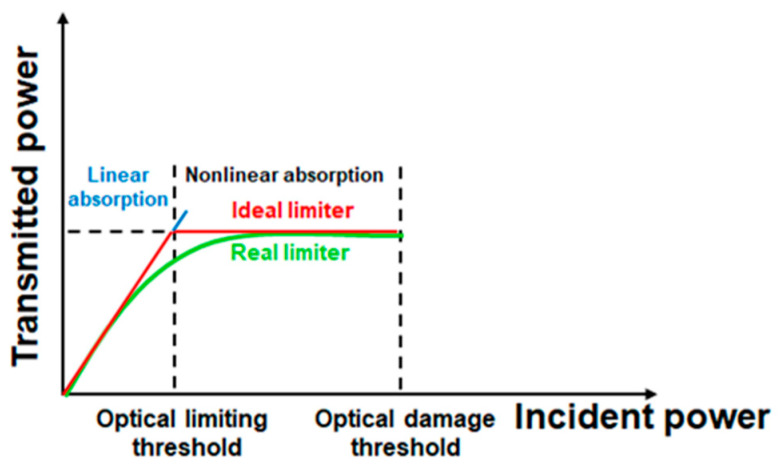
Optical limiting functionality.

One of the most promising practical applications of these biomaterials, based on the nonlinear absorption process is the optical limiting (OL) functionality. OL devices are of great practical importance for the protection of the human eye, light sensors, cameras, and other sensitive optical and optoelectronic devices against intense sources of laser radiation, which can irreversibly damage such elements when a safety damage threshold is exceeded [7,8,9,10,11,12,13]. The experimental dependence of the transmitted power/intensity on the same parameters of the incident beam is shown for an ideal OL (red line) and a real OL (green line), respectively (Figure 1). For the ideal OL, the transmitted power/intensity has a linear increase for incident power/intensity up to the OL threshold, remaining constant after this. For a real OL, the transmitted power/intensity depends nonlinearly on the incident one, being described by a saturation curve.

The continuous development of this type of device is crucially dependent on the availability of suitable optical materials. A wide variety of materials are being studied to achieve effective OL [11,14,15,16,17,18]. For example, in many papers, the OL functionality for various groups of materials, including organic dyes, other common organic materials, graphene, and its derivatives, fullerenes, polymeric materials (organic and/or inorganic), inorganic semiconductors, and other materials, has been extensively investigated in recent years [11,19,20,21,22]. At the same time, it has been shown that the synthetic polymers used so far in optical limiters can be successfully replaced by the biopolymers that have some advantages over synthetic materials [12]. Among the studied materials with optical limiting properties are carbon-based materials, e.g., graphene and its derivates [15,19,20,21,23]. The OL functionality of these materials, in different forms as solutions, films, and bulk, has been mainly studied for visible and near-infrared nanosecond and picosecond laser pulses [24,25,26,27,28] and, in a smaller measure, for femtosecond laser pulses at 800 nm wavelength [29,30,31,32,33]. Very few papers have investigated the OL of femtosecond laser pulses in the IR band, which includes the wavelength of 1550 nm, an important wavelength for communications. B. N. S. Sooraj et al. reported in a recent study [34] the optical limiting of 35 ps laser pulses at 532 nm in silver, gold, and alloy clusters, investigated by Z-scan technique. The authors demonstrate that Ag and Au clusters exhibit almost the same efficiency for optical limiting, but the alloy clusters are less efficient in optical limiting. Other materials with optical limiting properties are glasses doped with different nanoparticles [35,36]. Measurements of OL response in synthetic polymers (Ethylene propylene-diene monomer (EPDM), Poly(methyl methacrylate) (PMMA)) doped with dye (Disperse Red 1, Disperse Yellow, respectively) are also reported using 532 nm wavelength (10 ns pulses and continuous wave) [37,38]. However, DNA-based biopolymers, which have been intensively studied recently, present several advantages over materials based on synthetic polymers. Biomaterials generally present unique properties, which are not easily replicated in conventional organic or inorganic materials. In addition, the DNA biopolymer comes from renewable natural resources.

To the best of our knowledge, the OL properties of the DNA-based materials functionalized with natural dyes, have not yet been investigated and represent an innovative and original approach. The nonlinear optical investigation of this new class of biopolymer-based materials is complex and represents a topic of high interest in photonics. One of the most important reasons why DNA appears to be exciting for OL functionality is the exceptional capability of DNA chains to interact with various dyes (nonbinding interactions, groove binding, and intercalation/semi-intercalation) [39,40] and the suitability of its properties. Its particular, double-stranded helical structure shows that it can be used as scaffolding for incorporating functional molecules, allowing the development of photonic, electronic, and opto-electronic devices [6,22,39,40,41,42,43,44,45]. Moreover, there is a lot of free space to functionalize it with different dyes to obtain and tailor desired properties for targeted practical applications [4,5,22,39,40,41,42,43,44,45]. DNA can be used in fast photoinduced responses, improved nonlinear optical effects in DNA compounds [46,47], as well as in other future applications in nanotechnology [48,49,50]. Another important property of DNA in photonics is its large transparency range, from 350 to about 1700 nm, with a cutoff wavelength around 320 nm [22].

DNA is an eco-friendly biodegradable material that can be obtained at a low cost from renewable resources such as the waste produced by the food processing industry. Pure DNA is known to be only soluble in water and exhibits poor thermal stability (denaturation at around 90 °C), making it difficult to process into good optical-quality thin films. On the other hand, it is well known that the complex of DNA with cetyltrimethyl-ammonium chloride (CTMA) surfactant is soluble in alcohols and can be processed into thin films with good optical quality [44]. Moreover, in the past few years, it was discovered that this solubility allows the functionalization of DNA-CTMA with synthetic/natural dyes, which contributes to the enhancement of the nonlinear optical properties of the resulting complexes and tuning their sensitivities in different spectral ranges. Recently, it was shown that the DNA–surfactant complex represents an interesting medium for photosensitive molecules, with significantly lower kinetic chemical and photo-thermal degradation constants [24,51,52], as observed for synthetic polymers. The DNA-CTMA complex has high thermal stability up to a temperature of 100 °C [45], maintaining its double-stranded helical structure [53]. The DNA and DNA–surfactant complexes decompose in the 220–230 °C temperature range [40,53,54,55]. Their high thermal conductivity ensures a lower laser heating of materials based on them compared to other synthetic polymers (e.g., PMMA) with similar optical absorption [46]. Another advantage of this new class of materials is the increased optical damage threshold after functionalization with natural dyes compared to synthetic polymers, [12,13,23,40,54,55,56,57,58,59], showing that the biopolymers are more resistant to high-energy laser pulses than some synthetic polymers like PC or PEG [40,55].

In the present work, we experimentally demonstrate the OL functionality of a new class of nonlinear optical materials (NLO), namely DNA biopolymer functionalized with Turmeric dye, by I-scan experiments with ultrashort laser pulses (~120 fs) in NIR, at the important telecommunication wavelength of 1550 nm. To the best of our knowledge, the OL properties of these new class of NLO materials have not been investigated yet and are reported for the first time in the literature, representing an innovative and original approach. The influence of the DNA presence and the variation of the dye concentrations in the prepared solutions on the linear transmittance and the optical limiting performance of these novel materials are discussed. The values of the optical transmittance in the linear regime, the saturation intensity of the nonlinear transmittance curves, and the coefficient of the nonlinear absorption have been determined. The photostability and the thermal stability of the investigated solutions have also been evaluated by monitoring the temporal decay of the normalized absorption spectra under the illumination with UVA light and heating, respectively.

## 2. Materials and Methods

The investigated materials consisted of DNA biopolymer functionalized with Turmeric natural dye. To demonstrate the influence of the biopolymer on the optical limiting properties we also investigated materials without DNA. The DNA was purchased from Ogata Research Laboratory, Ltd., Chitose, Japan. DNA is an eco-friendly biodegradable material that exhibits very interesting properties for its use as a matrix for dye molecules, with many interesting applications in photonics [6,7,8,9,13]. It can be used to replace synthetic polymers with several advantages, which are due to its particular helical structure [53]. This biopolymer is extracted from the waste produced by the food processing industry and its sources are practically unlimited, making it an inexpensive biopolymer. The DNA used in this study is extracted from salmon waste. It has a high molar mass of ~8 MDa. The processing steps involved in obtaining DNA are presented in detail in [60,61]. In the preparation of samples investigated in this study, the DNA was used in the form in which it was purchased. The DNA was not sonicated, thus keeping unaffected its double-stranded helical structure. It is well known that sonication can destroy the double-stranded structure of DNA (ds-DNA) by breaking nucleobase bonds resulting in single-stranded deoxyribonucleic acid (ss-DNA). This alteration process of DNA depends on sonication conditions.

The unprotected DNA is soluble in water and its degradation time is quite short. With the CTMA surfactant, the DNA-CTMA complex becomes insoluble in water, but soluble in organic solvents, such as alcohols, and can be functionalized with different photosensitive dyes. The choice of CTMA as a surfactant for DNA functionalization is based on several reasons described in detail in [62]. The DNA–CTMA complex becomes water-insoluble and more stable mechanically due to the alkyl chain of CTMA. Also, the DNA-CTMA complex is soluble in organic solvents, such as alcohols, which makes easier the doping of the complex with different photosensitive molecules.

The DNA functionalization with CTMA was performed in this study following the procedure described by Grote et al. [60]. This procedure is briefly described below. DNA is negatively charged, soluble in water, and reacts with positively charged surfactants forming a stable DNA-CTMA complex through ionic chemical interaction. The DNA-CTMA complex is stable and insoluble in water, but soluble in several organic solvents, which facilitates its doping with dyes. The DNA-CTMA complex was obtained by dropwise addition of the DNA solution to the CTMA solution. The final solution is stirred for approximately 4 h after the DNA solution has been added and allowed to settle. The obtained precipitate complex is filtered using a filter and then washed well until the wash water contains no more traces of surfactant. The water is removed with another filter paper by manual pressing. The DNA-CTMA complex is dried in a desiccator under vacuum at 60 °C. The dry precipitate obtained is grinded until the particle size is small enough for its use in solutions. Next, the DNA-CTMA complex is doped with Turmeric, the link between them being ensured by an electrostatic interaction with Turmeric.

The natural extract of Turmeric was obtained by the maceration technique, a simple method by which ground raw material is kept in contact with the solvent for a defined time. The resulting product was mixed at 400 rpm for 24 h and then filtered through filter paper. The obtained solutions were placed in the refrigerator to avoid degradation. For the functionalization of the DNA-CTMA complex with Turmeric, we used butanol as an organic solvent, because it has several advantages over other solvents. It has a low vapor pressure of 6.7 hPa at 20 °C [63], compared to other alcohols, such as, for example, ethanol which has a pressure of 59 hPa at the same temperature of 20 °C, which ensures a relatively slow evaporation. Also, butanol’s moderate viscosity prevents evaporation [64]. The concentration of DNA-CTMA in butanol was 30 g/L. Solutions with different concentrations of Turmeric (3%, 5%, 7.5%, 10% and 15%) have been prepared. These concentrations represent the percentages of the Turmeric relative to the DNA-CTMA matrix.

In Table 1, we present the investigated solutions of DNA-CTMA-TURMERIC in butanol with different dye concentrations, and the solutions of Turmeric-natural dye in butanol, with similar dye concentrations.

### 2.1. Materials Characterization

The nonlinear optical properties are in strong correlation with the linear optical properties: the control of the linear optical properties can lead to a useful control of the nonlinear ones. The linear refractive index (*n*_0_) of the prepared solutions was determined using the ABBE refractometer (Carl Zeiss, Jena, Germany). The values of the refractive index are slightly dependent on Turmeric concentration. The refractive index of the solutions with the lowest concentration of Turmeric is *n*_0_ = 1.3969 for the T3 sample and *n*_0_ = 1.4018 for the T3-DNA sample. The refractive index of the solutions with the highest concentration of Turmeric is *n*_0_ = 1.3971 for the T15 sample and *n*_0_ = 1.4021 for the T15-DNA sample. The refractive indices of solutions with intermediate concentrations are in between the values corresponding to solutions with the lowest and highest Turmeric concentrations, respectively (the difference is only at the third decimal).

In the use of this novel DNA-based optical material, it is important that the light does not produce irreversible changes in its optical properties (photodegradation), and also its thermal stability. To investigate these aspects, we performed several spectral analyses (UV-VIS-NIR spectroscopy, before and after its UV illumination/heating) to check its photodegradation and thermal stability. These analyses provide us with valuable information about the photochemical/thermal stability of the novel compounds.

#### 2.1.1. UV-VIS-NIR Spectroscopy

**Figure 2:**
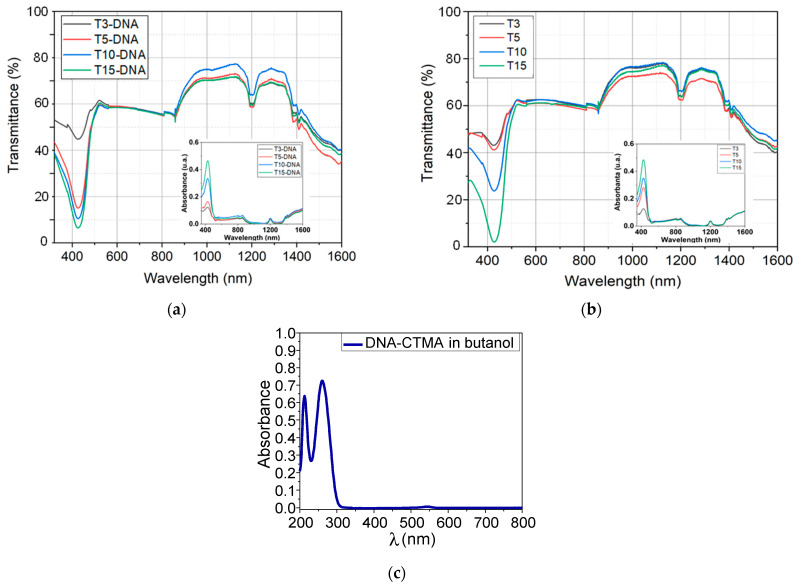
UV-VIS-NIR spectra of the DNA-CTMA-Turmeric solutions (**a**); Turmeric solutions (**b**), and DNA-CTMA solution (**c**) in butanol. (**a**) UV-VIS-NIR spectra of DNA-CTMA-Turmeric solutions in butanol. (**b**) UV-VIS-NIR spectra of Turmeric solutions in butanol. (**c**) UV-VIS-NIR spectra of DNA-CTMA solution in butanol.

The spectroscopic studies of the transmittance for the prepared solutions were done using a Perkin Elmer spectrophotometer (Perkin Elmer, Waltham, MA, USA), Lambda 950 model at a resolution of 1 nm. The UV-VIS-NIR transmission/absorption spectra of these innovative biomaterials, collected with air as reference were recorded between 300 and 1600 nm wavelengths. The recorded spectra are presented in Figure 2a and Figure 2b, respectively. The inset from Figure 2a,b shows the absorption bands of prepared solutions for different concentrations, at the same interval of wavelengths. The used spectrophotometric cell has a 0.5 mm optical path to avoid obtaining a saturated signal in the spectral range of interest

From the UV-VIS-NIR spectra, shown above in Figure 2a, it can be seen that the values of the transmission curves of T3, 5, 7.5, 10, and 15 solutions are 60–80% in the VIS-NIR range, while for solutions based on DNA functionalized with Turmeric dye, it is slightly lower than in the case of solutions without DNA, but remaining sufficiently high for the eye or sensitive imaging devices [14]. Thus, we can say that these materials are transparent enough to see through them, a requirement for passive optical limiting materials in NIR. The spectra confirm, as expected, the presence of Turmeric and DNA, from the existence of absorption peaks at wavelength of 420 nm, specific to the Turmeric dye (Figure 2a,b) [65], and of DNA-CTMA at wavelengths in 200–300 nm range (Figure 2c), respectively. The DNA-CTMA compound does not have any absorption peak in the 300–800 nm wavelength range, as can be seen from Figure 2c. The spectra have a quasi-constant transmittance in the visible range (500–850 nm), followed by a higher transmittance in the near-infrared range (850–1300 nm), excepting the dip at 1200 nm and by a lower and decreasing transmittance in the wavelength range of 1350–1500 nm.

Nevertheless, the transmittance does not drop below 40% in the considered VIS-NIR spectral range (500–1600 nm). As a general remark, a very slight decrease in the transmittance spectra can be observed in the case of solutions functionalized with DNA-CTMA compared to those without DNA; this fact does not impede the use of the DNA-CTMA-Turmeric compound in optical limiting. This difference may be due to the intercalation of the natural dye of Turmeric in the double-strand helical structure of DNA, which is an advantage that contributes to increasing the stability of the obtained DNA-CTMA complex. All these increase the resistance of solutions to photodegradation.

#### 2.1.2. Photostability of Prepared Solutions

**Figure 3:**
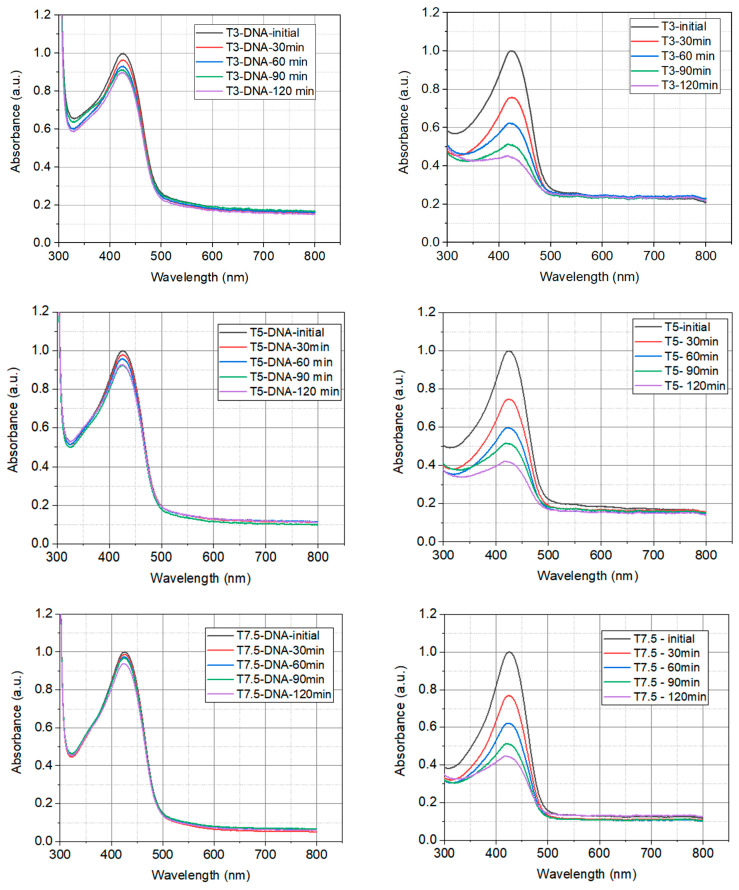

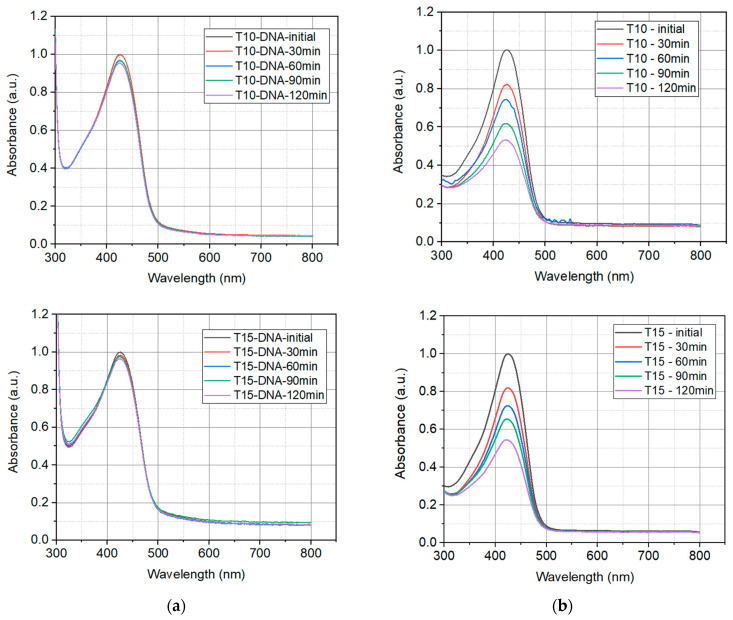
The evolution of the UV-VIS absorption spectra of the prepared solutions in butanol, under the influence of UVA irradiation at several exposure times: (**a**) DNA-CTMA-Turmeric (T3-DNA ÷ T15-DNA) and (**b**) Turmeric solutions in butanol (T3 ÷ T15).

The chemical stability of turmeric and DNA-CTMA-Turmeric complex in butanol exposed to UVA light has been studied to investigate the resistance of synthesized solutions to the action of a light source. The photodegradation of the synthesized solutions in butanol was carried out using the Luzchem Photoreactor—4× (Luzchem Research Inc., Ottawa, ON, Canada), equipped with 8 lamps, each with a power of 12 W. The natural extract was exposed to UVA irradiation at a wavelength of 325 nm. The photoreactor is equipped with a fan to maintain a constant temperature throughout the experiment. To perform the photodegradation experiments, constant volumes of the synthesized solutions and covered quartz cuvettes were used to avoid evaporation. The absorption spectra of the biopolymers in butanol were recorded both at the initial moment and after each exposure to irradiation. The absorption spectra were recorded using the Thermo Scientific Spectrophotometer (Fisher Scientific, Paisley, UK), Model 220. Figure 3 shows the overlays of the absorption spectra of investigated DNA-CTMA-Turmeric complexes with different concentrations and of the natural extracts of Turmeric without DNA (with the same concentrations) exposed to irradiation for different exposure times.

The absorption spectra acquired for each sample have been normalized to the maximum value of the amplitude which corresponds to the initial spectrum, recorded before the start of irradiation with UVA light. From the acquired spectrum, it can be seen that as the exposure time increases, the photodegradation of the Turmeric extract occurs much faster than the photodegradation of the DNA-CTMA-Turmeric, proved by a much higher decrease in absorbance. At the same time, increasing the concentration of Turmeric leads to a decrease in the photodegradation rate.

**Figure 4:**
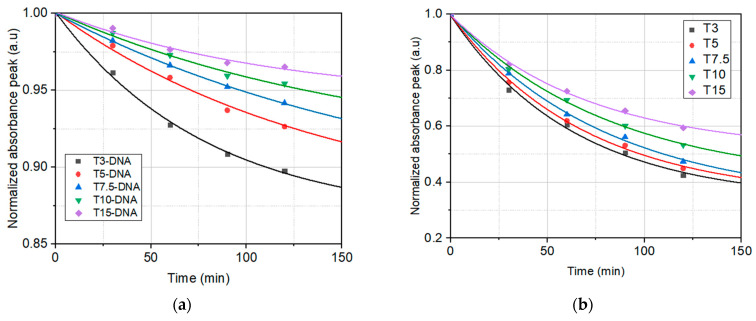
The temporal evolution of the normalized peak absorbance under the influence of UVA irradiation for: (**a**) DNA-CTMA-Turmeric solutions in butanol (T3-DNA ÷ T15-DNA) and (**b**) Turmeric solutions in butanol (T3 ÷ T15) (dots—experimental points; continuous lines—the fitting functions). The vertical scale is different in (**a**) with respect to (**b**).

The dependence on time of the peaks of normalized absorbance under the influence of UVA irradiation of the investigated solutions is shown for both sets of samples, T3-DNA ÷ T15-DNA and T3 ÷ T15, in Figure 4a,b, respectively. In this figure the experimental points, for all investigated solutions, have been fitted with exponential decay functions, $y_{Pi}(t)=y_{0Pi}+A_{Pi}\cdot \exp\left(-k_{Pi}\cdot t\right)$, *i* = 1, …, 5, shown as continuous lines.

The parameters *y*_0*Pi*_, *A_Pi_*, and *k_Pi_* of the fitting functions for the experimental data from Figure 4a,b, corresponding to the investigated solutions, are shown in Table 2.

This kind of mathematical function, which describes the temporal decay of the normalized absorbance peak of investigated solutions in butanol, could be a consequence of the degradation of the dye molecules in the excited volume of the sample, namely its decrease over time, due to their photodegradation induced by UVA light.

The fit of the temporal decay of the normalized absorbance peak of investigated solutions in butanol under the influence of UVA irradiation (Figure 4) reveals that the photodegradation rates are higher in solutions with only Turmeric, compared to the solutions containing DNA. This is proof of the higher photostability of Turmeric dye in the DNA-CTMA-Turmeric solutions in butanol.

The photodegradation studies have shown that the combined use of DNA-CTMA and natural extract of Turmeric results in a slight improvement in the photostability of DNA-CTMA-Turmeric compared to Turmeric only. Moreover, these complexes show higher chemical stability under UVA illumination than synthetic polymers, such as PMMA, under similar irradiation conditions [66,67].

#### 2.1.3. Thermal Degradation

Thermal analysis measurements were made to determine the thermal stability of Turmeric in butanol with/without DNA biopolymer.

**Figure 5:**
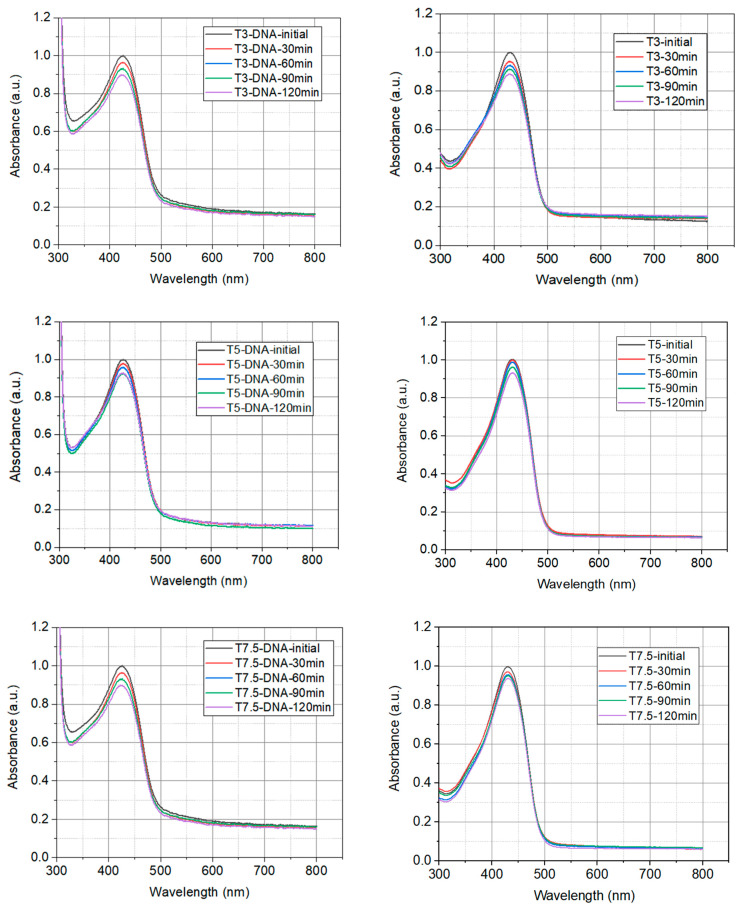

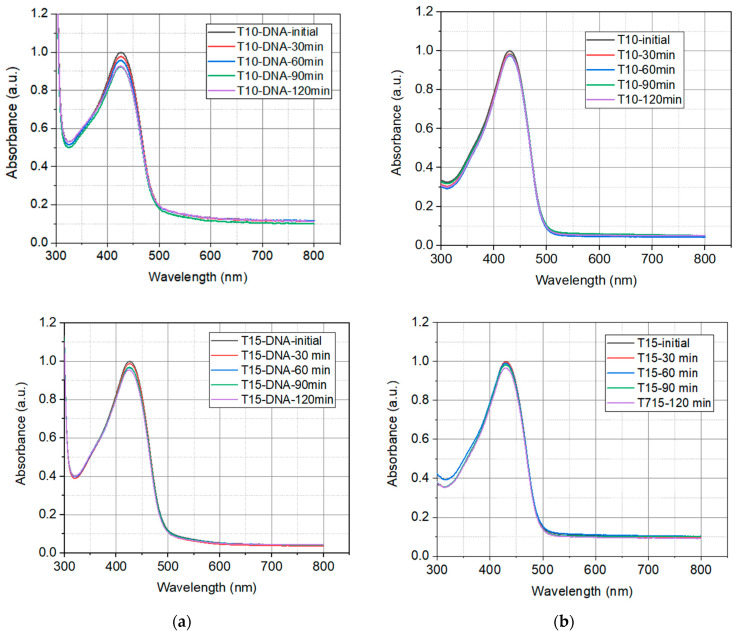
The evolution of the absorption spectra of the prepared solutions in butanol under the influence of heating at 60 °C, for several exposure times, (**a**) DNA-CTMA-Turmeric (T3-DNA ÷ T15-DNA) and (**b**) Turmeric solutions in butanol (T3 ÷ T15).

To test the thermal degradation several cycles of heating the samples for 30 min at 60 °C have been performed. The absorption spectra have been recorded, using a Thermo Scientific Model Evolution 220 spectrophotometer (Fisher Scientific, Paisley, UK), both at the initial moment and after each exposure interval. The obtained absorption spectra are shown in Figure 5.

**Figure 6:**
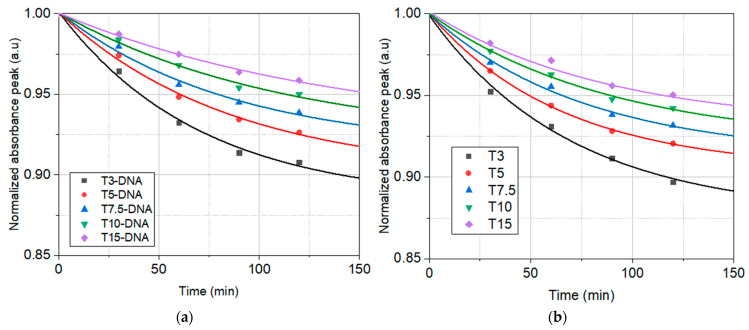
The temporal evolution of the normalized peak absorbance under the influence of heating at 60 °C for: (**a**) DNA-CTMA-Turmeric solutions in butanol (T3-DNA ÷ T15-DNA) and (**b**) Turmeric solutions in butanol (T3 ÷ T15) (dots—experimental points; continuous lines—the fitting functions).

The dependence on time of the peaks of normalized absorbance under the influence of heating at 60 °C of the investigated solutions is shown together for both sets of samples, T3-DNA ÷ T15-DNA and T3 ÷ T15, in Figure 6a,b, respectively. In this figure the experimental points for all investigated solutions have been fitted with exponential decay functions, $y_{Ti}(t)=y_{0Ti}+A_{Ti}\cdot \exp\left(-k_{Ti}\cdot t\right)$, *i* = 1, …, 5, shown as continuous lines.

The parameters *y*_0*Ti*_, *A_Ti_*, and *k_Ti_* of the fitting functions for the experimental data from Figure 6a,b, corresponding to the investigated solutions, are shown in Table 3.

The fit of the temporal decay of the normalized absorbance peak of investigated solutions heated at 60 °C (Figure 6) reveals that the differences between the thermal stability of the investigated solutions with and without DNA are much lower than the differences between their photostability (Figure 4). However, the thermal stability of DNA-CTMA-Turmeric solutions is slightly better than that of the Turmeric solutions. Thus, the analysis of the temporal decay of the normalized absorption spectra under illumination with UVA light and heating, respectively, revealed a better photo- and thermal stability of solutions containing DNA-CTMA.

## 3. Optical Limiting Capability: Measurements and Discussions

**Figure 7:**
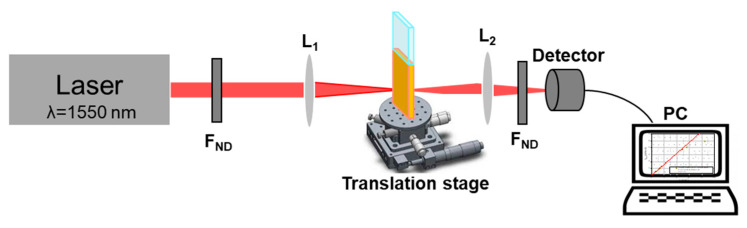
Schematic experimental configuration for optical limiting capability investigation by I-scan method.

The OL capabilities, dependent on the nonlinear optical properties of these new biomaterials, were explored by the Intensity scan (I-scan) technique [65,66]. The I-scan is a sensitive and powerful optical experimental method for the investigation of nonlinear optical refraction and/or absorption. A schematic of the I-scan configuration is shown in Figure 7.

In the I-scan measurements, we used as excitation source an Er-doped fiber laser (FemtoFiber Scientific FFS, TOPTICA Photonics AG, Munich, Germany) at the wavelength λ = 1550 nm, which generates ultrashort pulses about ~120 fs with a repetition rate of 76 MHz. The intensity incident on the sample is controlled with neutral density filters (F_ND_) (Thorlabs, Munich, Germany), which changes the laser power. All the filters used in the I-scan measurements are calibrated by us at the wavelength of 1550 nm. The incident beam on the sample has a maximum average power of ~193 mW. The corresponding peak power and the pulse energy of the generated laser pulses are ~20 kW and ~2.5 nJ, respectively. The average incident laser powers are chosen to be below the values for which the laser-induced damage could occur. The investigated biopolymers are placed in the focal plane of the lens L_1_ which has a focal length of 5 cm and focuses the incident beam to a spot with a diameter of 26 μm on the sample. The investigated samples are in the form of solutions and they have been placed in special demountable cuvettes, with optical quality windows made from quartz (Hellma, Müllheim, Germany) of 0.5 mm internal thickness and 130 μL internal volume. Their spectral domain of transparency is 200–2500 nm. These cuvettes were fixed on the micrometric translation stages for the fine-tuning of their positions. The power of both incident and transmitted beams has been corrected to Fresnel reflections on external (air–quartz) and internal (quartz–solution) interfaces. The L_2_ (focal length 6 cm) lens is used to adjust the spot size of the transmitted laser beam to the aperture of the detector which measures the beam’s average power. For precise adjustment of the lenses’ positions relative to the sample, we used micrometric translation stages (not shown in Figure 6). In front of the detector are placed different neutral density filters (F_ND_) to keep the power of the light incident on the detector, below its maximum measurable power. The incident and the transmitted beams were measured using the OP-2IR sensor (Coherent, Portland, OR, USA) coupled to a power meter (FieldMax II-TOP, Coherent, Saxonburg, PA, USA). No optical damage was observed in the investigated samples in the range of the laser peak intensities available in our experiments.

The I-scan method [68] was developed as a variant of the *Z*-scan technique, presenting a series of advantages compared to the classic *Z*-scan method [69]. In the case of the *Z*-scan method, during the movement of the sample along the focused light path (*Z* axis), the size of the illuminated area of the sample is dependent on its position with respect to the focal plane of the focusing lens, and the collected signal is averaged on areas of different sizes. This could be a problem in the case of inhomogeneous samples. In the I-scan method [68,69], the sample position on the optical axis of the system is fixed. The laser intensity on the sample is modified by neutral density filters (F_ND_).

The OL potential of the investigated DNA-based compounds was evaluated by measuring the transmitted peak intensities *I_trans_*(peak) of the samples as a function of the incident peak intensities *I_inc_*(peak). A deviation from the linear transmittance towards lower values is an indicator of optical limiting potential. This property of a nonlinear material is described by a linear transmission for low values of the incident light until a certain threshold value of the incident intensity is reached, after which the transmitted intensity remains constant when the incident intensity increases.

**Figure 8:**
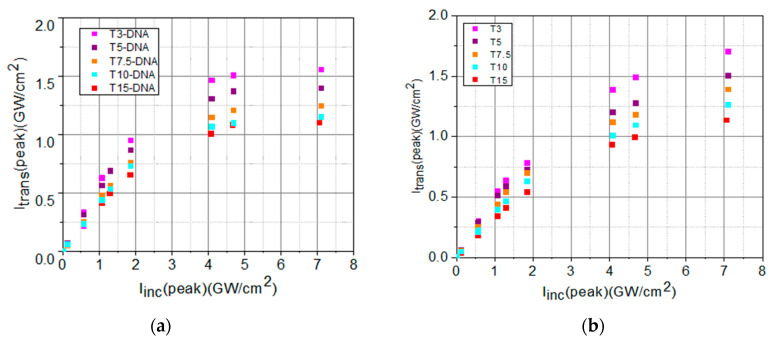
Transmitted peak intensities in function of the incident peak intensities for: (**a**) DNA-CTMA-Turmeric solutions in butanol (T3-DNA ÷ T15-DNA) and (**b**) Turmeric solutions in butanol (T3 ÷ T15).

The experimental results obtained during the investigation of the optical limiting capability of the biopolymer samples are shown and discussed below. The experimental peak intensities *I_trans_*(peak), of the beam transmitted through sample, in function of the peak intensities, *I_inc_*(peak), of the incident beam, are shown in Figure 8.

The values of the experimentally determined transmitted peak intensities, *I_trans_*(peak), for low incident peak intensities, *I_inc_*(peak), shown in Figure 8, have been fitted with a linear dependence described by the Lambert–Beer law, Equation (1):

(1) $$I_{trans,\;\;linear}=I_{inc}\cdot e^{-\alpha_{0}\cdot L}$$

**Figure 9:**
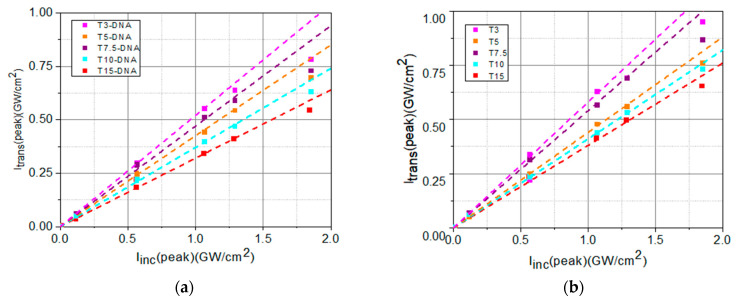
Transmitted peak intensities in function of the incident peak intensities, *I_inc_*(peak), for low incident intensities, fitted (dash lines) with a linear dependence (Equation (1)) for: (**a**) DNA-CTMA-Turmeric solutions in butanol (T3-DNA ÷ T15-DNA) and (**b**) Turmeric solutions in butanol (T3 ÷ T15).

From the slope of this linear dependence (Figure 9), we determined the values of the linear absorption coefficient, *α*_0_, of each sample.

A nonlinear dependence of *I_trans_*(peak) of the samples as a function of the incident peak intensities *I_inc_*(peak) indicates that the absorption coefficient is no longer constant, being dependent on incident intensity. In this case, Equation (1) becomes:

(2) $$I_{trans,\;NL}=I_{inc}\cdot e^{-\alpha \left(I_{inc}\right)L}$$

In our experiments, the dependence *I_trans_*(peak) of the samples as a function of the incident peak intensities *I_inc_*(peak) shows a saturation trend. We considered for α(*I_inc_*) from Equation (2) the following equation, in which the saturation intensity, *I_sat_*, and the nonlinear absorption coefficient, *β*, are considered [67]:

(3) $$\;\alpha \left(I_{inc}\right)=\frac{\alpha_{0}}{1+\frac{I_{inc}}{I_{sat}}}+\beta \cdot I_{inc}\;\;\;\;$$

**Figure 10:**
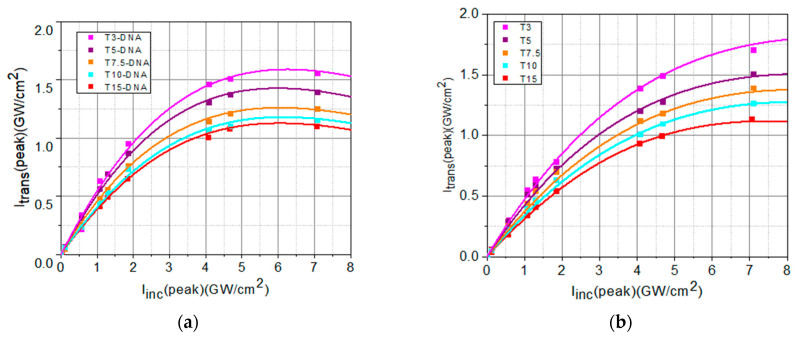
Transmitted peak intensities in function of the incident peak intensities, *I_inc_*(peak), fitted (continuous lines) with the nonlinear dependence given by Equation (2). for: (**a**) DNA-CTMA-Turmeric solutions in butanol (T3-DNA ÷ T15-DNA) and (**b**) Turmeric solutions in butanol (T3 ÷ T15).

The experimental data from Figure 8 have been fitted with de Equation (2), taking into account Equation (3), as shown in Figure 10. From the fit of the experimental data using Equations (2) and (3), the values of *I_sat_* and of *β* have been determined.

The values of linear and nonlinear optical parameters (the linear transmittance, *T_L_*, the linear absorption coefficient, *α*_0_, the saturation intensity, *I_sat_*, and the nonlinear absorption coefficient, *β*) determined by fitting the experimental data from Figure 9 and Figure 10 are summarized in Table 4.

**Figure 11:**
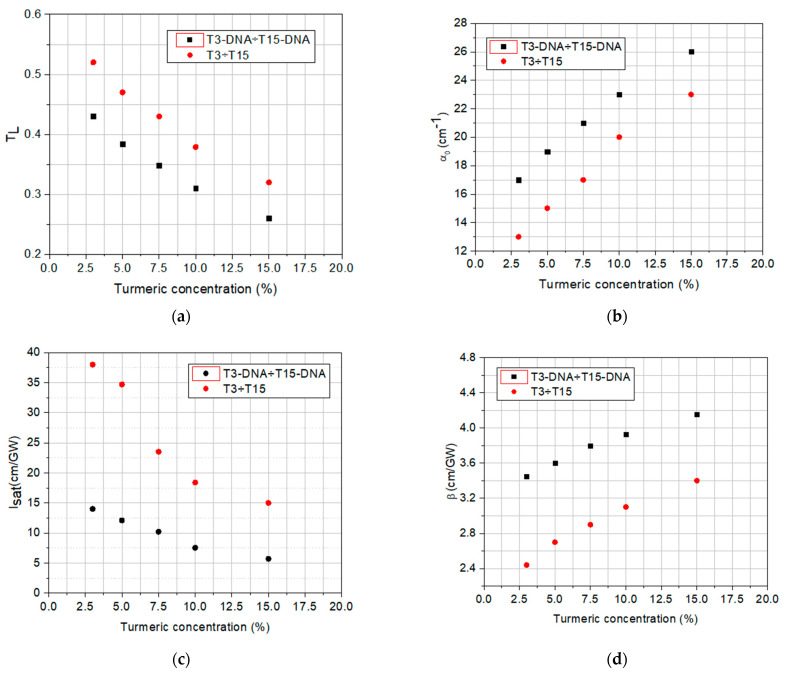
The dependences on Turmeric concentration of the linear transmittance, *T_L_* (**a**), linear absorption coefficient, *α*_0_ (**b**), saturation intensity, *I_sat_* (**c**), and the nonlinear absorption coefficient, *β* (**d**).

The analysis of the data from Table 4 and of their graphical representation from Figure 11 reveals the influence of the DNA-CTMA complex on linear and nonlinear optical absorption properties of the investigated materials, which are involved in the passive OL functionality.

It can be observed that the set of solutions with DNA-CTMA-Turmeric has a lower linear transmittance than the samples that do not contain DNA-CTMA. At the same time, the increase in the dye concentration decreases the linear transmittance. Also, the solutions containing DNA-CTMA have lower linear transmittances compared to solutions with only Turmeric. The range of absorption coefficient values can thus be extended/restricted to higher/lower values in the investigated compounds, with a tunability given by the dye concentration. The results reveal the relationship between the dye concentrations and the magnitude of the absorption coefficient.

For samples with different concentrations, the OL is stronger for those with lower *I_sat_.* Equation (2) provides information on the overall optical limiting capability of a sample, considering the nonlinear optical processes involved in this functionality. For samples with different linear transmittances, a larger deviation from the straight line (corresponding to linear transmission), at the same incident intensity, means better limitation.

The DNA-based matrix favorably influences the OL potential of the investigated DNA-CTMA-Turmeric samples, compared to Turmeric-only samples, in two different ways, one related to the OL process itself and the other one related to the photo- and thermal stability of the material. The performed OL experiments revealed that the OL process is more efficient in solutions containing DNA-CTMA, which is evidenced in Figure 9 and Figure 10, and Table 4. Thus, the deviation of the saturation-type fit of the experimental data from the straight line corresponding to linear transmittance is larger in samples with DNA-CTMA, where the transition from the linear regime to the limiting regime starts at lower values of incident intensity (Figure 9) and, consequently, the intensity range of optical limiting is broader. Moreover, the saturation intensity, *I_sat_*, is lower and the nonlinear absorption coefficient, *β*, is higher in these samples, as shown in Table 4 and Figure 11c,d. On the other hand, the results presented in Section 2.1.2 and Section 2.1.3 revealed increased photo- and thermal stability of samples with DNA-CTMA, which is important for OL functionality. Thus, the DNA-CTMA matrix is beneficial for the OL potential of Turmeric.

## 4. Conclusions

The optical limiting in solutions in butanol of DNA-CTMA-Turmeric compound and of Turmeric with similar concentrations has been experimentally demonstrated, for the first time to the best of our knowledge, by Intensity scan experiments with ultrashort laser pulses (~120 fs) in NIR at 1550 nm wavelength. The photostability and thermal stability of these solutions have also been evaluated. The analysis of the temporal decay of the normalized absorption spectra under illumination with UVA light and heating, respectively, revealed a better photo- and thermal stability of Turmeric in solutions containing DNA-CTMA.

The efficiency of the passive optical limiting caused by the nonlinear optical absorption in the above-mentioned novel materials has been comparatively evaluated. The analysis of the experimental data obtained in the I-scan experiment allowed the determination of some important optical parameters. Thus, from the analysis of the initial part of the experimental curves, when the material’s response is linear, the linear transmittance and the linear absorption coefficient have been determined. At higher incident intensities, the saturation trend of experimental curves and a nonlinear absorption have been considered. This analysis allowed us to determine the saturation intensity and the nonlinear absorption coefficient of the investigated materials, both of which are important for their OL capability. The study performed revealed a favorable effect of the DNA-CTMA matrix on OL in the investigated materials.

The obtained results are important in the applications of these novel DNA-based, eco-friendly materials for the protection of the human eye and sensitive devices against intense NIR laser radiation.

## Figures and Tables

**Table 1:** Denomination of the samples and Turmeric concentration in solutions.

Sample	Turmeric Concentration in Solution g/L
T3-DNA/T3	3
T5-DNA/T5	5
T7.5-DNA/T7.5	7.5
T10-DNA/T10	10
T15-DNA/T15	15

**Table 2:** The parameters *y*_0*Pi*_, *A_Pi_*, and *k_Pi_*, of the fitting functions for data points from Figure 4a,b.

	Film	T3-DNA/T3	T5-DNA/T5	T7.5-DNA/T7.5	T10-DNA/T10	T15-DNA/T15
Fitting Parameter						
*y*_0*Pi*_		0.8666/ 0.33452	0.87049/ 0.34271	0.87542/ 0.33081	0.90768/ 0.39744	0.94231/ 0.51004
*A_Pi_*		0.13429/ 0.66048	0.13029/ 0.65415	0.12578/ 0.66785	0.09363/ 0.59953	0.05685/ 0.48695
*k_Pi_* (min^−1^)		0.01259/ 0.01569	0.00969/ 0.01453	0.00721/ 0.01405	0.00698/ 0.01246	0.00645/ 0.01117

**Table 3:** The parameters *y*_0*Ti*_, *A_Ti_*, and *k_Ti_*, of the fitting functions for data points from Figure 6a,b.

	Film	T3-DNA/T3	T5-DNA/T5	T7.5-DNA/T7.5	T10-DNA/T10	T15-DNA/T15
Fitting Parameter						
*y*_0*Ti*_		0.8839/ 0.87714	0.9000/ 0.90468	0.9158/ 0.91292	0.92052/ 0.9204	0.92547/ 0.9310
*A_Ti_*		0.11726/ 0.12183	0.10091/ 0.0953	0.08525/ 0.08672	0.08021/ 0.7964	0.07502/ 0.07001
*k_Ti_* (min^−1^)		0.0141/ 0.01512	0.01159/ 0.01449	0.01151/ 0.01299	0.00882/ 0.01111	0.00703/ 0.00943

**Table 4:** The linear transmittance, *T_L_*, the linear absorption coefficient, *α*_0_, the saturation intensity, *I_sat_*, and the nonlinear absorption coefficient, *β*, determined from the fit of the experimental data (Figure 9 and Figure 10).

Sample	Linear Transmittance	*α*_0_ (cm^−1^)	*I_sat_* (GW/cm^2^)	*β* (cm/GW)
T3-DNA/T3	0.43/0.52	17/13	14/38	3.5/2.4
T5-DNA/T5	0.38/0.47	19/15	12/35	3.6/2.7
T7.5-DNA/T7.5	0.36/0.43	21/17	11/21	3.8/2.9
T10-DNA/T10	0.31/0.37	23/20	7/18	3.9/3.0
T15-DNA/T15	0.27/0.32	26/23	6/16	4.0/3.4

## Data Availability

Data are contained within the article.
